# Fast alternating projection methods for constrained tomographic reconstruction

**DOI:** 10.1371/journal.pone.0172938

**Published:** 2017-03-02

**Authors:** Li Liu, Yongxin Han, Mingwu Jin

**Affiliations:** 1 School of Electronics and Information System, Tianjin University, Tianjin, People’s Republic of China; 2 Department of Physics, University of Texas Arlington, Arlington, Texas, United States of America; Chongqing University, CHINA

## Abstract

The alternating projection algorithms are easy to implement and effective for large-scale complex optimization problems, such as constrained reconstruction of X-ray computed tomography (CT). A typical method is to use projection onto convex sets (POCS) for data fidelity, nonnegative constraints combined with total variation (TV) minimization (so called TV-POCS) for sparse-view CT reconstruction. However, this type of method relies on empirically selected parameters for satisfactory reconstruction and is generally slow and lack of convergence analysis. In this work, we use a convex feasibility set approach to address the problems associated with TV-POCS and propose a framework using full sequential alternating projections or POCS (FS-POCS) to find the solution in the intersection of convex constraints of bounded TV function, bounded data fidelity error and non-negativity. The rationale behind FS-POCS is that the mathematically optimal solution of the constrained objective function may not be the physically optimal solution. The breakdown of constrained reconstruction into an intersection of several feasible sets can lead to faster convergence and better quantification of reconstruction parameters in a physical meaningful way than that in an empirical way of trial-and-error. In addition, for large-scale optimization problems, first order methods are usually used. Not only is the condition for convergence of gradient-based methods derived, but also a primal-dual hybrid gradient (PDHG) method is used for fast convergence of bounded TV. The newly proposed FS-POCS is evaluated and compared with TV-POCS and another convex feasibility projection method (CPTV) using both digital phantom and pseudo-real CT data to show its superior performance on reconstruction speed, image quality and quantification.

## Introduction

Iterative image reconstruction methods have shown many advantages over the conventional analytic reconstruction methods, such as sophisticated imaging system modeling and easy incorporation of prior information for increased image quality and/or reduced radiation dose. In X-ray computed tomography (CT), to deal with data inconsistence caused by noise or insufficient data such as compressed-sensing sampling, two major constrained iterative reconstruction frameworks are extensively investigated: unconstrained optimization [1–9] and constrained optimization [10–16].

The unconstrained optimization is in line with maximum *a posteriori* (MAP) estimation, which combines the data fidelity and regularization terms together for a single unconstrained objective function and balances their contributions using a weighting parameter. For example, the Gauss-Seidel algorithm was applied to solve the reconstruction with either Gaussian or non-Gaussian priors [1, 3]. A conjugate-gradient preconditioning method was proposed to achieve faster convergence rate for gradient-based iterative reconstruction [2]. Recently, the advance in applying variable splitting approaches and Nesterov’s momentum techniques in image processing has resulted in novel fast algorithms for image reconstruction [6–9]. There is also active interest in developing advanced regularization terms (priors) beyond the pioneer total variation (TV) [11, 14], such as normal-dose image induced TV [17], total generalized variation [18], TV-stokes [19], *l*_*0*_ gradient [20], Haralick texture measures[21]. However, the unconstrained optimization methods usually face the challenge of choosing the appropriate weighting parameter to balance the data fidelity and regularization terms.

In contrast, constrained optimization uses either data fidelity or image properties, such as TV, as the objective function and puts others into constraints. It is often more straightforward in such a way that reconstruction parameters can have physical meanings and be determined from the data, e.g. the error bound of the data fidelity term in constrained TV minimization. For constrained optimization, alternating projection methods have pioneered successful reconstruction for few-view and limited-angle CT data [11, 14]. These methods typically combine projection onto convex sets (POCS) for data fidelity and nonnegative constraints with TV minimization, a.k.a. TV-POCS. Although TV-POCS is easy to implement and effective, there are several drawbacks: 1) it may converge to the mathematical optimum, but not the physical optimum (see Section “Rationale of using convex sets of feasibility instead of the constrained optimization” for more details); 2) the convergence of the method is lack of analysis and is sensitive to empirically selected parameters; and 3) the gradient descent method for (smoothed) TV minimization is generally slow [11–14].

In this work, we focus on addressing the issues with TV-POCS and propose a more efficient and accurate solution for constrained optimization of CT image reconstruction by using 1) the concept of convex feasibility; 2) alternating projection/POCS; and 3) a primal-dual hybrid gradient descent (PHGD) method [22] for TV projection. Convex feasibility approaches are to find feasible solutions of CT reconstruction that fall in convex sets [15] in case that the imaging system is highly ill-posed and the single optimum is elusive. In this work, these convex sets are defined to satisfy the conditions of 1) data fidelity, 2) non-negativity, and 3) bounded TV function, although other proper definitions can be used as well. The data fidelity is usually enforced by algebraic reconstruction techniques (ART) and the non-negativity by forcing negative values as zero. For the bounded TV function, we use the PHGD method [22], which was shown to be faster than state-of-the-art methods for the TV-*l*_2_ image denosing problem, such as fast iterative shrinkage thresholding (FISTA), Nesterov, alternating direction method of multiplier (ADMM), and Chambolle-Pock primal-dual algorithms [23]. It is noted that the Chambolle-Pock method has been adapted to solve the convex feasibility projection problem with the TV constraint (CPTV) for image reconstruction [15, 16] that is closely related the proposed method. Thus, we focus on comparing our method with TV-POCS using gradient descent [11, 14] and CPTV using projection onto *l*_1_ ball in the image gradient space [16] since all three methods can be generalized under the hood of vector space projection and solved by a full sequential alternating projection or POCS (FS-POCS). We also show that gradient-based methods can converge under a certain condition in the framework of FS-POCS and provide a theoretical way to safeguard parameters to meet the convergence condition. These developments lead to a theoretically sound and efficient iterative reconstruction. Furthermore, the proposed FS-POCS method can be easily extended to CT reconstruction with other types of data fidelity and image constraints as long as the convexity is satisfied.

The paper is organized as follows: the principle of the proposed method is described in section Methods, along with the rationale of convex feasibility projection and the introduction of the closely-related methods for comparison. The experiments and results are presented in the following section. The last two sections are devoted to Discussion and Conclusions, respectively.

## Methods

### CT imaging model and reconstruction with constrained TV minimization

X-ray CT imaging can be represented by a simplified linear model as,

p=Mx+e, (1)

where **pϵ**R^M^ is projection data (i.e. logarithm of inversely normalized detected X-ray photon fluence), **Mϵ**R^M×N^ is the system matrix that describes CT imaging, **xϵ**R^N^ is the image of linear attenuation coefficients to be reconstructed, and **e** is noise. The CT reconstruction can be expressed as constrained optimization as follows:

$$x=\arg\;\underset{x}{\min}{\left\|x\right\|}_{TV}$$ (2)

$$\mathrm{subjectto:}x=\arg\;\underset{x}{\min}{\left\|x\right\|}_{TV}$$ (3)

andx≥0, (4)

where ‖•‖ is the *l*_2_ norm in data domain, ‖•‖_*TV*_ is the TV function in image domain (${\left\|x\right\|}_{TV}=\sum_{u,v}\left|{\left(\nabla x\right)}_{u,v}\right|$), which is denoted as TV(**x**) as well in the following context, and ε is the squared error caused by the noise and imperfect image modeling.

### Rationale of using convex sets of feasibility instead of the constrained optimization

**Fig 1:**
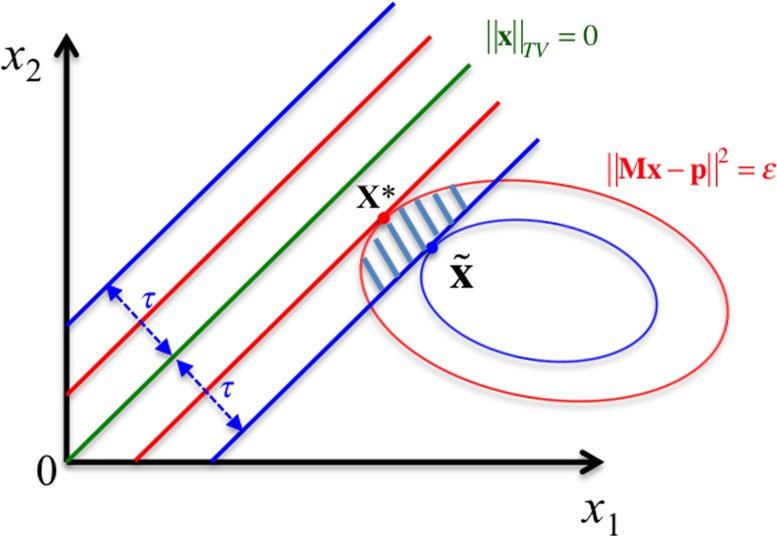
Different solutions for TV constrained CT reconstruction. The constrained TV minimization has a solution at the red point x*. If assuming the true image a TV value of τ ≥ *TV*(x*), the optimal solution should be at the blue point. The shaded area represents the convex feasibility solutions, which could produce better image and save computation.

The minimization of TV function in Eq 2 under the constraints of the data fidelity in Eq 3 and non-negativity in Eq 4 originates from the compressed sensing theory [24, 25], where the sparsity of image gradient space represented by TV function is enforced to achieve high-quality image reconstruction with few-views or limited-angels of projection data [11, 14]. As these three functions are convex, a global optimal solution is existed and can be obtained through convergent iterative methods, such as efficient first order methods [5, 23]. As shown in Fig 1 (for an easy conceivable 2-D case), the global optimal solution of constrained TV minimization, x* (the red dot in Fig 1), will lie on the boundary of ‖**Mx** − **p**‖^2^ = ε, which produces the minimal TV value in a non-trivial case (In a trivial case, the minimal TV value is zero when the uniform image is inside the ellipsoid region of ‖**Mx** − **p**‖^2^ ≤ ε).

Although the constrained optimization leads to a unique solution with the minimized TV value, the underlying true image may not attain such a low TV value. If the true image indeed has a TV value τ ≥ *TV*(x*), the optimal solution would be at the blue dot $\tilde{x}$ in Fig 1. As the TV value of the true image is unknown, a relaxed TV value may be used either based on some prior information or derived from large data sets and lead to a feasible solution set shown in the shaded region in Fig 1 (Note that in this case τ represents estimated TV upper bound that is greater than the true TV value, and thus the physical optimal solution $\tilde{x}$ is in the shaded area). With this treatment, two advantages can be achieved: 1) the solution is close to the physical optimum (inside the shaded area); and 2) POCS can be very fast to converge to a convex set, while converging to a single point is often slow due to collinearity, where the convex sets’ boundaries are often tangent to each other.

### Convex sets of feasibility and proximal mapping

For the reasons stated in 2.2, we replace the TV minimization by the following constraint,

$${\left\|x\right\|}_{TV}\leq \tau ,$$ (5)

The constrained CT reconstruction is then converted to find a solution that satisfies the three constraints of Eqs 3, 4 and 5, which define three convex sets in R^N^ space. The data fidelity term, Eq 3, defines points in R^N^ that are transformed to the R^M^ space and have a summed Euclidean distance to all hyperplanes no greater than $\sqrt{\varepsilon }$. It can be understood as an *l*_2_ spherical ball with a radius of $\sqrt{\varepsilon }$ in R^M^ space. Eq 4 is the nonnegative constraints confining in positive orthant and boundaries. Eq 5 is the TV constraints representing in an *l*_1_ diamond ball of image gradient with a radius of τ. The reconstruction goal is to find the image that falls into the intersection of the three convex sets (in consistent cases, i.e. *C*_0_ = *C*_1_ ∩ *C*_2_ ∩ *C*_3_ is not empty) or somewhere closest to them (in inconsistent cases, i.e. *C*_0_ = *C*_1_ ∩ *C*_2_ ∩ *C*_3_ is empty) [16]. It can be expressed as the following optimization:

$$x*=\arg\;\underset{x}{\min}\left\{\sum_{i=1}^{3}\delta_{C_{i}}(F_{i}(x))\right\},$$ (6)

where the sets *C*_1_, *C*_2_, and *C*_3_ satisfying the constraints defined in Eqs 3, 4 and 5, respectively, are convex in R^N^ space and *F*_*i*_ are affine transform functions. The indicator function is defined as $\delta_{C_{i}}(y)=\{\begin{array}{c} 0,\mathrm{if}y\in C_{i}\\ \infty ,\mathrm{if}y\notin C_{i} \end{array}$. Given any arbitrary initial image or intermediate image **x**, the proximal mapping to the convex set *C*_*i*_ is defined as:

$$\begin{array}{cc} \begin{array}{l} \mathrm{prox}(x)=\arg\;\underset{x}{\min}\left(\delta_{C_{i}}(F_{i}(x))+\frac{1}{2}{\left\|y-x\right\|}^{2}\right)\\ \;=\arg\;\underset{x\;\mathrm{with}\;y\in C_{i}}{\min}{\left\|y-x\right\|}^{2}\\ \;=P_{C_{i}}(x) \end{array} & \mathrm{for}i=1,2,3. \end{array}$$ (7)

where $P_{C_{i}}(x)$ represents the Euclidean projection onto each convex set *C*_*i*_, i.e. from any start point, a feasible solution is a point in set *C*_0_ that has the closest Euclidean distance to the start point. Such a solution can be solved using POCS, i.e. enforcing Euclidean projections on *C*_1_, *C*_2_ and *C*_3_ either sequentially or simultaneously. The POCS will converge to the solution as the R^N^ Euclidean space is a Hilbert space [26].

### Projection onto convex sets (POCS)

Using convex sets of feasibility, a solution of the set *C*_0_ can be achieved by the sequential projections onto *C*_1_, *C*_2_, and *C*_3_, respectively, denoted as P_C1_, P_C2_ and P_C3_ [26]. The details of these projections are given as follows.

*1)* *Projection onto C*_*1*_*—P*_*C1*_

Due to the huge size of **M**, the projection onto *C*_1_ is achieved using iterative methods. The noise free data fidelity set, $C_{1}^{'}=\left\{x:{\left(Mx-p\right)}^{T}(Mx-p)=0\right\}$, is a subset of set C_1_. It is equivalent to the linear equation **Mx** = **p** defining M hyperplanes in R^M^. The projection onto the *i*^th^ hyperplane can be achieved by the following equation,

$$P_{C_{1i}}(x)=x+\frac{1}{{\left\|m_{i}\right\|}^{2}}(p_{i}-m_{i}x)m_{i}^{T},\mathrm{for}i=1,\ldots ,M,$$ (8)

where **m**_*i*_ is the *i*^th^ row of **M** and *p*_*i*_ is the *i*^th^ element of **p**. Eq 8 is well-known algebraic reconstruction techniques (ART). The ART can also be performed in parallel to speed up the reconstruction, such as simultaneous algebraic reconstruction technique (SART) [27]. The projection onto set *C*_1_ can be obtained by conducting ART until the summed Euclidean distance in R^m^ is less or equal to ε. In real implementation, due to the computationally intense forward and backward projections, each projection onto one hyperplane is treated as one step of POCS and *P*_*C1*_ is defined as *P*_*C11*_*P*_*C12*_*…P*_*C1M*_ of M projections before moving to *P*_*C2*_ and *P*_*C3*_. Note that the ART in Eq 8 is used for projection *P*_*C1*_ in this work although other iterative methods can be used as well.

*2)Projection onto C*_*2*_*—P*_*C2*_

The projection onto a nonnegative set is straightforward and given in the following equation,

$$P_{C_{2}}(x)=\{x|x_{j}=\max\;\{x_{j},0\}\;\mathrm{for}\;j=1,2,\ldots ,N\}.$$ (9)

*3)Projection onto C*_*3*_*—P*_*C3*_

Given a starting point **v** that is outside of *C*_3_ (i.e. the image after *P*_*C1*_ and *P*_*C2*_ in the proposed algorithm), the projection onto set *C*_3_ to satisfy the TV constraint is the nearest point to **v** on *C*_3_, i.e.

$$P_{C_{3}}(x)=\arg\;\underset{x}{\min}{\left\|x-v\right\|}^{2}\;\mathrm{s.t.}\;TV(x)\leq \tau ,$$ (10)

where $TV(x)\equiv {\left\|x\right\|}_{TV}$. Eq 10 is equivalent to the classical TV denoising problem although here the image TV value needs to be no greater than τ instead of approaching the minimum. It can be solved using either the projection onto the diamond ball in the gradient space of the reconstructed image [28] or first order methods for TV minimization [13, 22, 23].

In this work, the Lagrange method is used to derive *P*_*C3*_ so that first order methods can be applied accordingly. The projection in Eq 10 can be wrote as minimizing the Lagrange function

$$P_{C_{3}}(x)=\arg\;\underset{x}{\min}J(x)=\arg\;\underset{x}{\min}\left[{\left\|x-v\right\|}^{2}+\alpha (TV(x)-\tau )\right],$$ (11)

where the Lagrange multiplier *α* is a positive constant, whose range can be determined as follows.

Assuming **x**_*p*_ is the projection of **v** onto *C*_3_, i.e. *TV*(**x**_*p*_ = τ and *J*(**x**_*p*_) = min *J*(**x**), this leads to

$$J(x_{p})=\delta^{2}+\alpha (TV(x_{p})-\tau )\leq \alpha (TV(v)-\tau )=J(v),$$ (12)

where δ = ‖**x** − **v**δ. Since *TV*(**x**_*p*_) − τ = 0, Eq 12 can be simplified as

$$\alpha (TV(v)-\tau )\geq \delta^{2},$$ (13)

If the TV function is replaced by it smooth version (for gradient based optimization, see Eq 17 below), the smoothed TV function satisfies the Lipschitz inequality [5],

$$\left\|TV(x_{p})-TV(v)\right\|\leq L_{TV}\delta ,$$ (14)

where *L*_*TV*_ is the Lipschitz constant of the smoothed TV, which can be determined by Eq 3.5) in [5] and was set as 80 in this work. Combining Eqs 13 and 14, we have

α(TV(v)−τ)≥δ2≥‖TV(v)−τ‖2LTV2. (15)

It means that the Lagrange multiplier *α* must satisfy the following condition,

α≥‖TV(v)−τ‖LTV2. (16)

(On the other hand, $\alpha \leq \frac{(2-\mu )\delta^{2}}{{\left(\nabla TV(v)-\nabla TV(x_{p})\right)}^{T}(x_{p}-v)}$ if *J*(**x**) is strongly convex with strong convexity parameter *μ*.) We thus set *α* to be the lower bound of Eq 16, and determine *P*_*C3*_ by minimizing Eq 11.

The minimization of Eq 11 is a standard Rudin, Osher, and Fatemi (ROF) TV denoising problem with a given constant of *α* [29]. It can be solved iteratively using existing algorithms such as the gradient descent [5, 16], the conjugate gradient [30] and the first order primal dual methods [22, 31, 32]. When the conventional gradient needs to be calculated for the projection onto the TV set (e.g. TV-POCS), the smooth version of the original TV norm can be represented by the Huber function,

$$H_{\gamma }({\left\|\nabla x\right\|}^{2})=\{\begin{array}{l} {\left\|\nabla x\right\|}^{2}/(2\gamma ),\mathrm{if}\;\left\|\nabla x\right\|\leq \gamma \\ \left\|\nabla x\right\|-0.5\gamma ,\mathrm{else} \end{array},$$ (17)

where *γ* is a small number [5, 14].

In the proposed FS-POCS, we enforce *P*_*C3*_ using a first order primal-dual hybrid gradient (PDHG) method in this study [22]. Specifically, the minimization problem Eq 11 can be rewritten as a primal-dual formulation:

$$\underset{x\in R^{N}}{\min}\underset{y\in Y}{\max\;}\;\Phi (x,y)=x^{T}Ay+\frac{1}{\alpha }{\left\|x-v\right\|}^{2},$$ (18)

where a dual variable **y** is introduced that satisfies $TV(x)=\underset{y\in Y}{\max\;}x^{T}Ay$ ($A\in R^{N\times 2N}$is the discrete version of the negative divergence operator and $Y=\{y:y=\left[\begin{array}{c} y_{1}\\ y_{2}\\ \vdots \\ y_{N} \end{array}\right]\in R^{2N},y_{j}\in R^{2},\left\|y_{j}\right\|\leq 1\;\mathrm{for}\;j=1,2,\ldots ,N\}$) and the constant term −ατ is omitted from the optimization process. Given any intermediate solution (**x**^*k*^, **y**^*k*^) at iteration step *k*, the PDHG updates the solution following alternated dual and primal steps:

i) Dual step: fix **x** = **x**^*k*^, apply one step of gradient ascent method to the maximization problem $\underset{y\in Y}{\max\;}\Phi (x^{k},y)$ along the ascent direction $\nabla_{y}\Phi (x^{k},y)=A^{T}x^{k}$, **y**^*k*^ is updated as

$$y^{k+1}=P_{Y}(y^{k}+\beta \frac{2}{\alpha }A^{T}x^{k}),$$ (19)

where *P*_*Y*_ is the projection onto the set *Y* and *β* is the dual step size.

ii) Primal step: fix **y** = **y**^*k*+1^, apply one step of gradient descent method to the minimization problem along the descent direction $-\nabla_{x}\Phi (x,y^{k+1})=-(Ay^{k+1}+\frac{2}{\alpha }(x^{k}-v))$, **x**^*k*^ is then updated as

$$x^{k+1}=x^{k}-\theta (\frac{\alpha }{2}Ay^{k+1}+x^{k}-v),$$ (20)

where *θ* is the primal stepsize. Note that the convergence of PDHG is insensitive to the choice of step sizes (*β*, *θ*). Simple fixed values of (*β*, *θ*) can give satisfactory results although adaptive ones may further increase the convergence rate. In this work, we used fixed values: 2 for *β* and 0.2 for *θ*. Please refer to [22] for more details of PDHG.

It was shown in [16] that PDHG is among the top performers in solving the ROF problem. It is worth noting that the gradient descent method used in TV-POCS [14] can also lead to the projection onto *C*_3_ if certain conditions are satisfied (see **S1 Appendix**).

Once we defined projections *P*_*C1*_, *P*_*C2*_ and *P*_*C3*_, **x** = (*P*_*C3*_*P*_*C2*_*P*_*C1*_)**x** can be conducted repeatedly. Based on the theory of vector space projection, the sequence converges weakly to a point in convex set *C*_0_ if *C*_0_ is non-empty (or strongly in a finite-dimensional space) [26].

### FS-POCS reconstruction algorithm

We thus propose a reconstruction algorithm by enforcing projections onto set C_1_, C_2_, and C_3_ sequentially. We call this method as full sequential POCS (FS-POCS). The details of the algorithm are listed below:

1. Initialize image **x**, data fidelity error bound *ε*, and TV bound *τ*
2. Conduct projection *P*_*C1*_ (Eq 7) and *P*_*C2*_ (Eq 8)
3. Conduct projection *P*_*C3*_
  - Determine *α* using Eq 16
  - Conduct projection *P*_*C3*_ (Eq 11) using the PDHG method
4. Repeat step 2) and 3) until the change of images in two successive iteration is less than a predefined value.

The method proposed in [33] is used to determine the parameter *ε* as the sum of the projection noise variance among all projection bins. The parameter *τ* can be determined either from prior images, as the same type of images will generally have similar TV values, or from less accurately reconstructed images with a scaling factor for desired TV values. These parameters are physical meaningful and can be determined independently from reconstruction algorithms, especially when large data sets exist, while the parameter used in unconstrained optimization methods leveraging the balance of data fidelity and prior knowledge is algorithm-dependent and hard to determine beforehand.

### Existing similar methods

#### TV-POCS [11]

TV-POCS [11] falls in the framework proposed in this work. It solves the constrained CT reconstruction problem in two stages: POCS for the two constraints in Eqs 3 and 4, and TV minimization in Eq 2. Similar to FS-POCS, the two constraints in TV-POCS are achieved through projections *P*_*C1*_ and *P*_*C2*_ represented by Eqs 8 and 9, respectively. The TV minimization is achieved by using the gradient descent of TV function,

$$x^{k+1}=x^{k}-\eta s^{k},$$ (21)

where $s^{k}=\frac{\nabla TV(x^{k})}{\left\|\nabla TV(x^{k})\right\|}$ is the normalized gradient of smoothed TV function and *η* is the step size to assure non-increasing of TV function. Denoting *ρ* as a small positive number and *d_A_* as the sum of the absolute difference between images of POCS and TV minimization stages, *η* is determined by η = ρ*d_A_*.

The step size is a key parameter to achieve desired solution in TV-POCS methods, which was empirically selected in [14]. To better balance the data fidelity and TV terms, an adaptive steepest descent method was proposed, in which *ρ* becomes a variable and is updated according to image changes during iterations [11]. Another adaptive approach to determine this parameter based on image iterations were proposed in [12]. As shown in the **S1 Appendix**, the gradient descent of TV minimization can lead to a non-increasing *J*(**x**) if the step-size satisfies the following condition:

$$0\leq \eta \leq T^{k}=2\left(\langle 2(x^{k}-v),s^{k})\rangle +\alpha \left\|\nabla TV(x^{k})\right\|\right)/L.$$ (22)

#### CPTV [16]

In this work, we form CT reconstruction problem as a convex feasibility problem similar to the CPTV method [16]. In [16], the reconstruction problem was casted as a primal minimization problem, where the objective function is composed of the Euclidean distance between the start image and the reconstructed image and two indicator functions of data fidelity and *l*_1_ norm of the gradient tensor image. Instead of directly solving the primal problem, the dual problem was formed and the solution was found by minimizing the primal and dual gap. The major difference of our work from CPTV [16] is: 1) POCS is used in this work to get the solution sequentially, whereas in [16] the Chambolle-Pock primal-dual method was used to solve a unified primal minimization and dual maximization problem, where the constraints were expressed as indicator functions; 2) the projection onto the TV constraint set is achieved by PDHG in this work, whereas in [16] the TV constraint was satisfied by projection onto the *l*_1_ diamond ball of the gradient tensor image; and 3) the proposed method is remarkably simpler than CPTV, yet achieves faster convergent reconstruction, as shown in the following results. It is worth noting that CPTV may not be computationally efficient, but is useful for algorithm prototyping.

## Experiments and results

We investigated the proposed method using digital phantom and pseudo-real CT data. The performance of the proposed FS-POCS method was then compared to the existing methods, filtered backprojection (FBP), TV-POCS and CPTV. We also validated that the TV-POCS method satisfies the convergence condition of gradient descent methods shown in Eq 22. All iterative reconstruction methods ran 1,000 times and the reconstructed image size was 256x256, unless otherwise stated. The same reconstruction parameters as the original publications were used for TV-POCS [11] and CPTV [16], while for FS-POCS *ε* was calculated as the sum of the projection noise variance among all projection bins (1.79 for the Shepp-Logan data; 0.8717 for the pelvis phantom data; and 1.4682 for the cadaver head data) and *τ* was calculated from the ground-truth images (152 for the Shepp-Logan data; 298 for the pelvis phantom data; and 723 for the cadaver head data), unless otherwise stated.

### Numerical experiment settings

#### Shepp-Logan phantom

**Fig 2:**
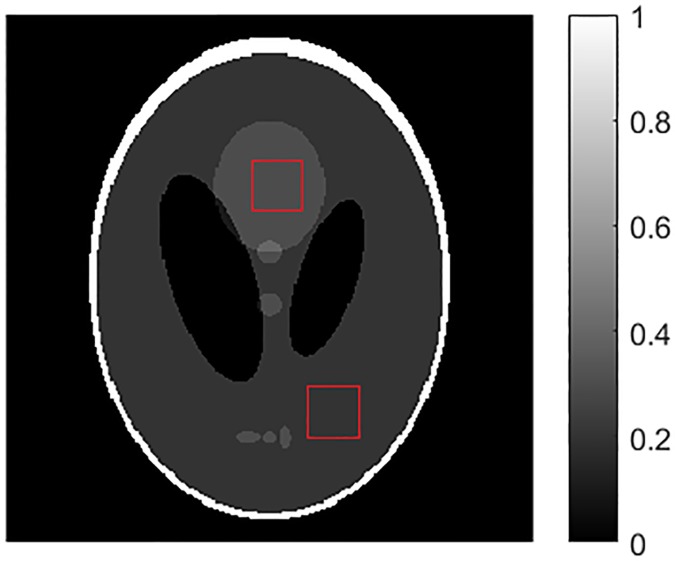
The Shepp-Logan Phantom. The regions of interest (ROIs) in red squares are for CNR calculation.

In the simulation experiments, a 256x256 Shepp-Logan phantom with 1mm^2^ pixels is used. Its background is filled with water equivalent material with a linear attenuation coefficient of 0.02 mm^-1^ at 80 KeV. The projection data were collected using the fan beam scanner with 720 bins under the Poisson noise assumption. The pitch of bins was set to 1mm. The source to detector distance (SDD) was 800mm, and the source to iso-center distance (SAD) was 400mm. The projection data of the phantom were generated with different number of projection views evenly distributed along a circular orbit. The radiation intensity was set to 5.0x10^5^ counts per incident ray. Quantitatively, we use two metrics for reconstruction image quality: root mean square error (RMSE) between the reconstructed image and the ground truth image and contrast to noise ratio (CNR) of the region of interest (ROI). The Shepp-Logan phantom image (using linear attenuation coefficient mm^-1^) is shown in Fig 2. The regions marked with a red rectangle are the ROIs selected for CNR measure = (mean of the bright region–mean of the dark region)/(standard deviation of the dark region). These two measures reflect the global and local reconstruction accuracy, respectively.

Although the digital Shepp-Logan phantom data provide benchmark evaluations for FS-POCS, it is instructive and practically meaningful to assess its capability of reconstructing more complex structures of physical phantoms and real human anatomy. In this regard, we tested different reconstruction methods on two sets of pseudo-real data as described below.

#### Pseudo-real data 1 –An anthropomorphic pelvis phantom

The slice images of an anthropomorphic pelvis phantom (CIRS 801-P) (Computerized Imaging Reference Systems Inc., Norfolk, VA) were acquired by a multi-detector CT (MDCT) (Phillips Brilliance Big Bore, Philips Healthcare, Nevada, US). The images (512x512x198) at 1 mm^3^ resolution were reconstructed from 1,000 projection views per rotation using the vendor built-in reconstruction algorithm. The first set of pseudo-real data was then generated by projecting one slice image into 60 views evenly over 360 degrees using a fan-beam scan with 1.0x10^5^ photon counts per incident ray.

#### Pseudo-real data 2 –Cadaver head

The second set of pseudo-real data was generated using the real human male cadaver head CT data from the Visible Human Project (http://www.nlm.nih.gov/research/visible/getting_data.html). The selected head volume has a dimension of 256 x 256 x 245 and a voxel size of 1 mm^3^. Its slices were re-projected into 60 views over 360 degrees using a fan-beam scan with 1.0x10^5^ photon counts per incident ray.

### The influence of TV priors for reconstruction

**Fig 3:**
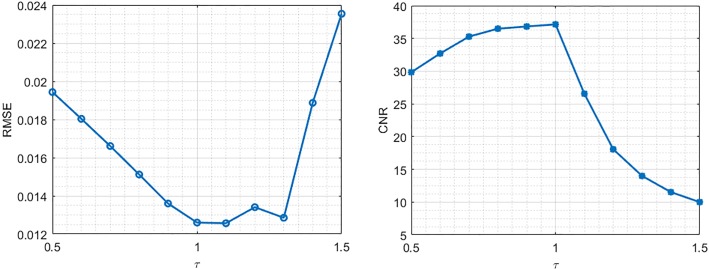
TV prior values’ influence on reconstruction accuracy. RMSE (a) and CNR (b) value changes with the different TV bounds ([0.5 0.6 0.7 0.8 0.9 1.0 1.1 1.2 1.3 1.4 1.5] times of the true TV value *τ*.

The TV value of the original Shepp-logan phantom is *τ* = 152. In this experiment, we chose TV bounds equal to [0.5 0.6 0.7 0.8 0.9 1.0 1.1 1.2 1.3 1.4 1.5] times of *τ*, and then applied FS-POCS to reconstruct images, respectively. The RMSE and CNR in the ROI were calculated and plot versus the TV bounds in Fig 3. As can be seen, the best RMSE and CNR values are achieved at the TV bound equal to the ground truth 1.0 *τ*. It is also noted that the behavior of the global measure RMSE is not exact as that of the local measure CNR. This experiment demonstrates that the TV value does not necessarily to attain the lower value in order to recover the image closest to the ground truth image.

### Comparison of different methods for Shepp-Logan phantom

**Fig 4:**
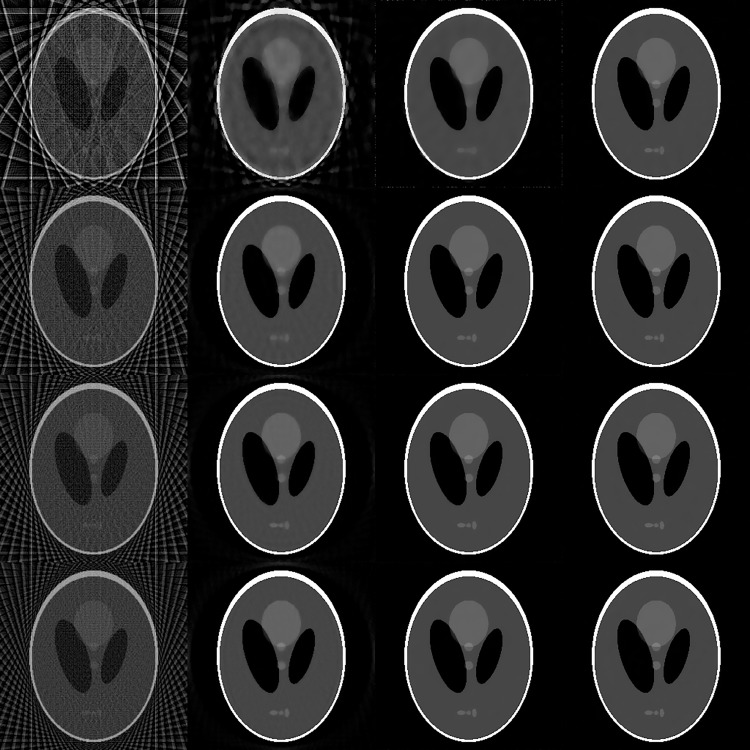
Reconstructed images from data of different projection views for Shepp-Logan phantom. From left to right: FBP, CPTV, TV-POCS and FS-POCS; from top to bottom: 24, 48, 60, 72 projection views.

Beside the proposed FS-POCS method, the filtered back-projection (FBP), CPTV and TV-POCS methods were also applied for reconstruction. The reconstructed images are shown in Fig 4 for comparison. The results of iterative methods demonstrated better quality than the FBP method in all experiments. More details are preserved on images of the TV-POCS and FS-POCS at 48 and 60 views. Compared with the other methods, FS-POCS is notably superior at the 24 views.

**Fig 5:**
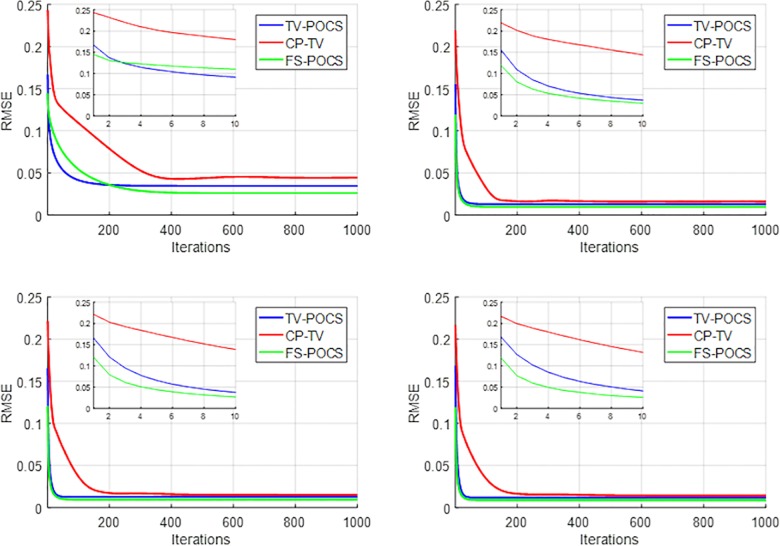
RMSE of reconstructed images changing with iterations for data with different projection views. Top left to right: 24, 48 views; bottom left to right: 60, 72 views. The inserts are the first ten iterations.

**Fig 6:**
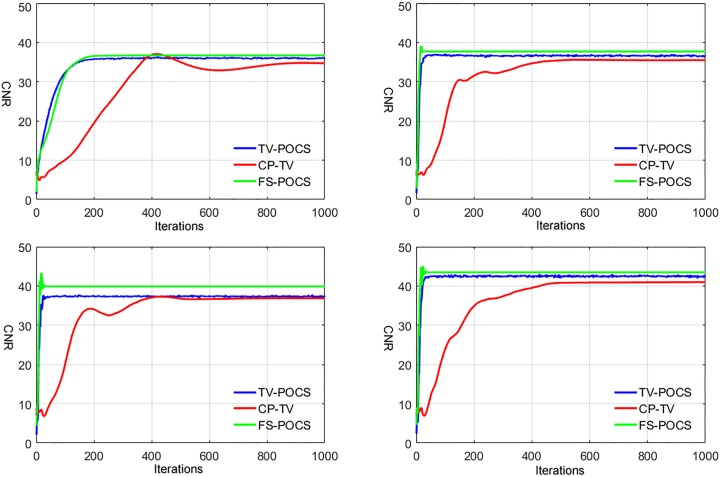
CNR of Reconstructed images changing with iterations for data with different projection views. Top left to right: 24, 48 views; bottom left to right: 60, 72 views.

The quantitative measures of RMSE between the ground truth image and the reconstructed images and CNR in selected ROIs changing along the iteration numbers are plotted in Figs 5 and 6, respectively, for TV-POCS, CPTV and FS-POCS (with the optimal prior 1.0 *τ* value). As can be seen, FS-POCS converges either comparable to or much faster than other iterative methods and usually attains better reconstruction results (lower RMSE and higher CNR). This reflects that the mathematic optimal (approached by TV-POCS) may not be the physical optimal as shown in Fig 1 and FS-POCS solutions could be closer to the physical optimal than TV-POCS and CPTV. Again, the behavior of the global measure RMSE is not exact as that of the local measure CNR.

The computational time for 1,000 iterations is shown in Table 1 for different reconstruction methods. Each method was implemented in MATLAB on a computer using an Intel Core I7-770 CPU with 32GB memory. FS-POCS takes much shorter time than TV-POCS and CPTV due to the fewer TV minimization steps. TV-POCS suffers from the same 20 TV minimization sub-iterations. Combined with the faster convergence rate (i.e. fewer iterations for convergence shown in Fig 5), FS-POCS can significantly shorten the total reconstruction time.

**Table 1:** The computational time for 1,000 iterations of each method in seconds.

# of projection views	TV-POCS	CPTV	FS-POCS
24	38.38	14.38	9.84
48	42.90	20.24	15.77
60	48.47	23.77	18.87
72	52.17	25.19	21.94

### Numerical behavior of TV-POCS and FS-POCS

In this part, we investigate the convergence of TV-POCS by analyzing the change of *J*(**x**) during TV minimization and the corresponding step size change along the threshold *T*^*k*^. The reconstruction with 60 views is conducted using TV-POCS described in [14] while keeping step-size of gradient descent satisfying Eq 22.

**Fig 7:**
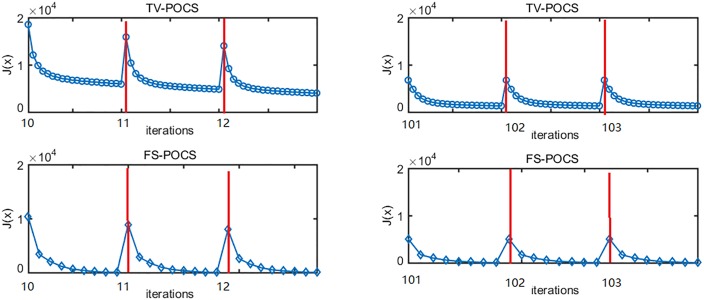
Changes of *J*(x) with TV-POCS and FS-POCS iterations.

The changes of the distance function *J*(**x**) with TV-POCS iterations are shown in Fig 7A, during 10^th^ to 12^th^ main iterations (left) and during 101^st^ to 103^rd^ iterations (right). Between two successive main iterations, the TV gradient descent sequence is shown to demonstrate the change of *J*(**x**). In all these cases, *J*(**x**) decreases in each TV gradient-descent subroutine. Thus, Eq 21 is effective to achieve *P*_C3_. FS-POCS is also shown for comparison in Fig 7B. First, the iteration number of *P*_C3_ in FS-POCS is controlled by τ value and much smaller than fixed twenty TV minimization iterations used in TV-POCS. Second, the iteration number of FS-POCS decreases along the iteration (eight for 10^th^ to 12^th^ main iterations and seven for 101^st^ to 103^rd^ main iterations). Finally, the *J*(**x**) values are generally lower for FS-POCS compared to TV-POCS at the same main iteration.

**Fig 8:**
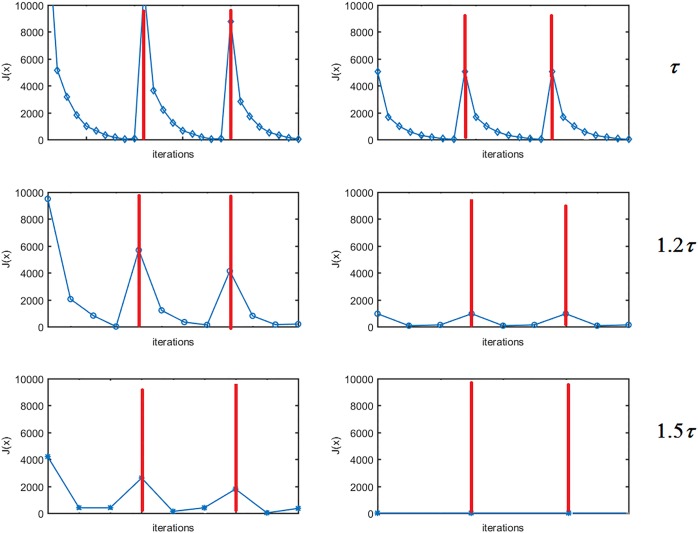
Changes of *J*(x) of FS-POCS with different TV bounds. From top to bottom: 1.0 *τ*, 1.2 *τ*, 1.5 *τ*.

It is also noted that the oscillation between *P*_C3_ and other two projections, even though the amplitude of the oscillation decreases along the iteration. The small size oscillation may be caused by the numerical precision, but the non-negligible oscillation is likely due to the tight selection of the parameters *ε* and *τ*, which results in an empty intersection. To avoid the oscillation, either the parameters *ε* and *τ* can be adjusted to be have a non-empty intersection, or simultaneous POCS can be used to reach the unique solution even for an empty intersection, which is the closest point to all constraint sets. One example using FS-POCS is shown in Fig 8, where the TV bound is gradually relaxed from the top to the bottom. It is observed that when the TV bound is sufficient large (1.5 *τ*), the oscillation diminishes as the iteration goes on. However, the caveat is that such a solution without oscillation may not necessarily be the closest to the physically optimal solution.

**Fig 9:**
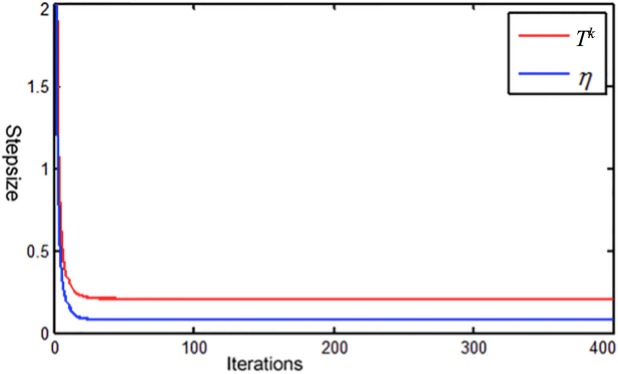
Changes of step size *η* and the corresponding threshold *T*^*k*^ in the TV-POCS method.

As shown in Fig 9, the step-size *η* is always smaller than *T*^k^ during the whole iteration and satisfied the convergence condition of Eq 22. Therefore, we observed the convergence behavior in Fig 7A. However, the smaller value of the step-size than *T*^*k*^ may lead to slower convergence.

### Results for pseudo-real data

**Fig 10:**
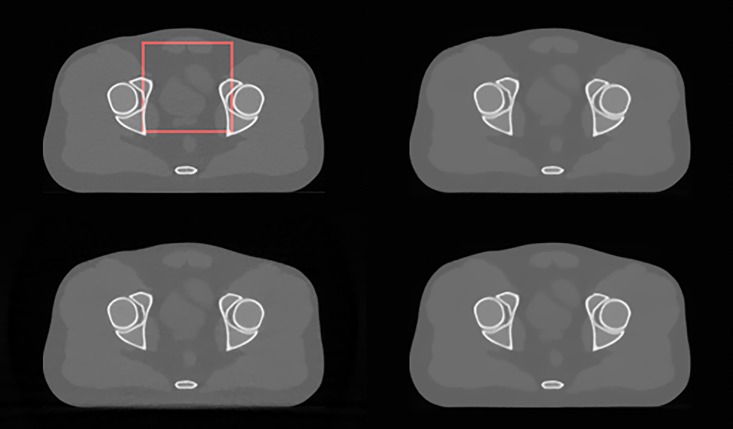
Original and reconstructed images for the pelvis phantom data. The original MDCT image of the pelvis phantom is at top left, and reconstructed images using 60 projection views are for TV-POCS (top right), CPTV (bottom left) and FS-POCS (bottom right), respectively. The red square denotes the ROI for enlarged view in Fig 11.

**Fig 11:**
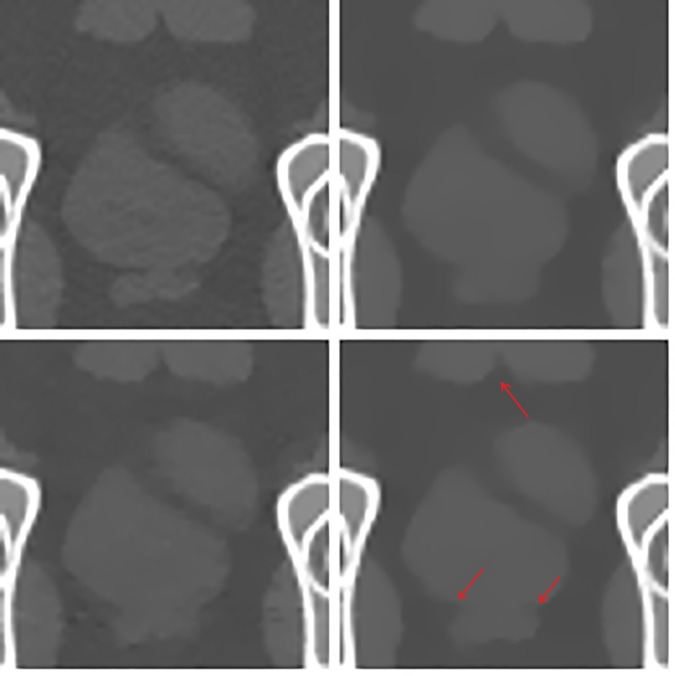
The magnified ROI images of the original MDCT (top left), TV-POCS (top right), CPTV (bottom left) and FS-POCS (bottom right).

In Fig 10, the original image of the pelvis phantom from MDCT (top left) is shown along with images reconstructed using 60 projection views for TV-POCS (top right), CPTV (bottom left) and FS-POCS (bottom right). All images look similar, especially for the high contrast bony structures. For the low contrast ROI (red rectangle in Fig 10), the enlarged view is shown in Fig 11. It is clearer that the original MDCT image keeps the fine structure best due to the use of full view data, but suffers notable noise because of filtered backprojection reconstruction used by the vendor MDCT. For the few-view data, the proposed FS-POCS method maintains the low contrast structure better than TV-POCS and CPTV, e.g. the areas indicated by the arrows. CPTV seems to suffer most blocky artifacts.

**Fig 12:**
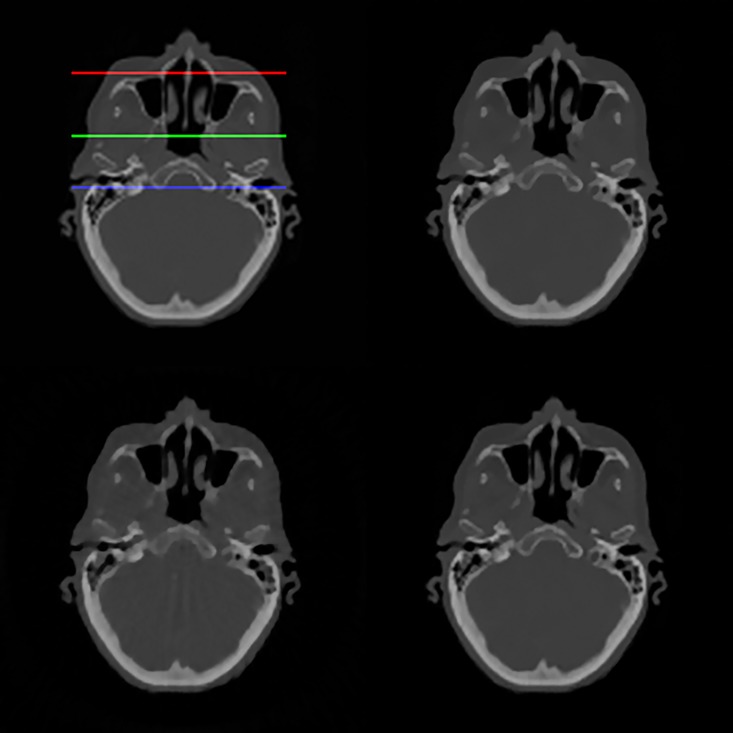
Original and reconstructed images for the cadaver head data. The original image from the Visible Human Project is at top left and reconstructed images from 60 views are for TV-POCS (top right), CPTV (bottom left) and FS-POCS (bottom right). The three lines are for the line profiles in Fig 13.

**Fig 13:**
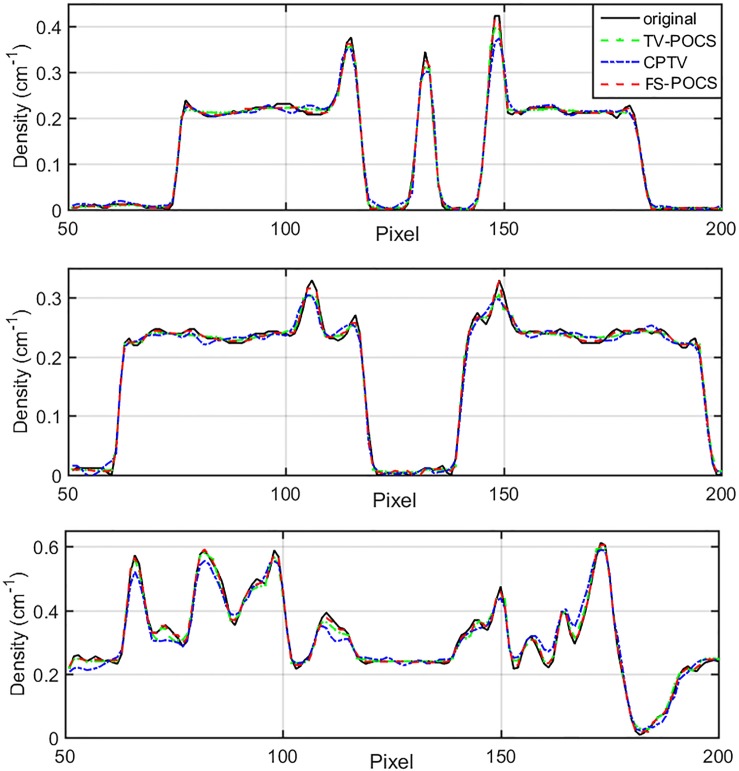
Line profiles of cadaver head images using different reconstruction methods (dashed lines) compared with that of the original image (solid lines).

The original cadaver head image from the Visible Human Project is shown in Fig 12 (top left) and the three lines represent the locations for the line profiles of reconstructed images shown in Fig 13. The images reconstructed from 60 views are shown in Fig 12 for TV-POCS (top right), CPTV (bottom left) and FS-POCS (bottom right). CPTV presents notable artifacts. TV-POCS and FS-POCS look similar to each other. The detailed difference is shown in the line profiles in Fig 13. It is subtle, but discernable that FS-POCS (red line) achieves the closest line profiles to the original ones (black line).

## Discussion

In this work, we treat the constrained CT reconstruction as a convex-set feasibility problem and propose an alternating projection algorithm, FS-POCS, to find the solution. Due to the uncertainty of the data fidelity error bound and the TV value of the original image, the rationale behind convex-set feasibility is that the mathematical optimal point of the constrained optimization may not be the same as the physical optimal point as shown in Fig 1 theoretically as well as the experimental results in Fig 3. In this regard, the proposed FS-POCS framework may find a solution of closer to the ground truth image than the constrained optimization problem. In addition, the advantage of two-stage methods is that the optimization problem is composed of two (major) separate parts: 1) *P*_*C1*_ for data fidelity constraint and 2) *P*_*C3*_ for the TV constraint. The improvement of the computational complexity can be greatly simplified through this separation and lead to faster computation. For instance, due to use of the projection and backprojection operations, ART is generally more computational intense than TV minimization. Two-stage algorithms usually perform more TV minimization steps than ART steps for a good balance of reconstruction performance and computational speed [11–14]. Furthermore, the relaxed projection algorithms (*P_relaxed_* = (1 − λ)**x** + λ*P***x** for λ ∈ (0, 2)) may be used for faster POCS convergence. Finally, the initial starting point may affect the final solution and the convergence speed in theory since the convex feasibility approaches seek solutions falling into the union of a set of convex constraints, instead of a unique point. For the phantom and pseudo-real data used in this study, we conducted further investigations on FS-POCS using different starting points (all zeros, all 0.2, random (uniformly distributed in [0 1]), and all ones) at the noise level of 5x10^4^ photons per incident ray. Although the initial convergence speed is different, after 400 iterations, all of them converge to the very similar result. It demonstrates that FS-POCS is robust to the choice of the initial points, at least for the data used in this study. The solution of FS-POCS is determined by the parameters ε and τ as shown in Fig 1. Since 03C1ε is determined by the noise level of data, prior knowledge of the physical constraining parameters, such as τ, shall be included as much as possible, either from the patient previous images [17, 34] or from a large repository of high-quality images with similar anatomical and pathologic structures [35].

We also derive the convergence condition if the gradient descent of TV minimization is used. If this condition is satisfied, the TV-POCS method can be adapted to find the solution similar to that of the convex-set feasibility problem even though it is not as efficient as FS-POCS, which uses an adaptive number of iterations to satisfy the TV constraint. A specific convergence condition on the step size of gradient descent can be exploited to assure the convergence for two-stage algorithms instead of heuristic parameter tuning [11–13].

The optimization problems of image reconstruction can take various forms that take into account the imaging model, the noise model, and desired physical properties of image objects. In this work, we focus our effort on developing an efficient and accurate reconstruction framework FS-POCS, which resolve the complicated optimization problem of image reconstruction into simple projections onto convex sets. Not only can the fast convergence be achieved, but also the reconstruction performance can be well controlled by physically meaningful parameters instead of the weighting parameters used in unconstrained optimization, where trial-and-error is usually needed to choose the suitable ones. As more advanced regularization constraints have been vigorously pursued [17–21], FS-POCS can provide a powerful and easy tool to efficiently reconstruct images with desired properties if the constraints are convex or can be well approximated by a convex projection.

Finally, as the early stage of the development of FS-POCS, we focused on high X-ray photon counts cases, i.e. low noise cases, since the least-squares criterion (Eq 3) works well in these cases, where Gaussian distribution is a good approximation of noise. To further shed light on its performance at different noise levels, we tested FS-POCS for 1x10^4^ photons per incident ray (high noise), 5x10^4^ photons per incident ray (medium noise), and 1x10^5^ photons per incident ray (low noise) using Shepp-Logan phantom. The proposed algorithm can reconstruct satisfying images for all case, with only a slight deterioration at extremely few views (24 views) and high noise (1x10^4^ photons per incident ray). Nevertheless, the caveat is that for high noise data, the weighted least-squares criterion [3, 13] or even more advanced noise models may need to be used as well as more advanced regularization constraints [17–21].

## Conclusions

In this work, we proposed a convergent convex feasibility based reconstruction framework, FS-POCS. It relies on physically meaningful parameters instead of the arbitrary weighting parameter used in unconstrained optimization. The experimental results on digital phantom and pseudo-real CT data demonstrated the superior image quality, quantitative results and convergence speed. FS-POCS can potentially shift the dependence on empirical parameters to that on physical parameters for iterative CT reconstruction. Further investigation is in plan to demonstrate the effectiveness of FS-POCS on real patient data.

## Supporting information

S1 Appendix Convergence of *J*(x) using the gradient descent of TV function. (DOCX)

## References

[1] Sauer K. and Bouman C., "A local update strategy for iterative reconstruction from projections," IEEE Transactions on Signal Processing, vol. 41, pp. 534–548, 1993.

[2] Fessler J. A. and Booth S. D., "Conjugate-gradient preconditioning methods for shift-variant PET image reconstruction," IEEE transactions on image processing, vol. 8, pp. 688–699, 1999 10.1109/83.760336 18267484

[3] Wang J., Li T., and Xing L., "Iterative image reconstruction for CBCT using edge-preserving prior," Medical Physics, vol. 36, pp. 252–60, 2009 10.1118/1.3036112 19235393 PMC2739313

[4] Defrise M., Vanhove C., and Liu X., "An algorithm for total variation regularization in high-dimensional linear problems," Inverse Problems, vol. 27, p. 065002, 2011.

[5] Jensen T. L., Jørgensen J. H., Hansen P. C., and Jensen S. H., "Implementation of an optimal first-order method for strongly convex total variation regularization," BIT Numerical Mathematics, vol. 52, pp. 329–356, 2012/06/01 2012.

[6] Kim D., Ramani S., and Fessler J. A., "Combining Ordered Subsets and Momentum for Accelerated X-Ray CT Image Reconstruction," IEEE Trans Med Imaging, vol. 34, pp. 167–78, 1 2015 10.1109/TMI.2014.2350962 25163058 PMC4280323

[7] Niu T., Ye X., Fruhauf Q., Petrongolo M., and Zhu L., "Accelerated barrier optimization compressed sensing (ABOCS) for CT reconstruction with improved convergence," Phys Med Biol, vol. 59, pp. 1801–14, 4 7 2014 10.1088/0031-9155/59/7/1801 24625411 PMC4080804

[8] Ramani S. and Fessler J. A., "A splitting-based iterative algorithm for accelerated statistical X-ray CT reconstruction," IEEE Trans Med Imaging, vol. 31, pp. 677–88, 3 2012 10.1109/TMI.2011.2175233 22084046 PMC3298196

[9] Li J., Niu S., Huang J., Bian Z., Feng Q., Yu G., et al, "An Efficient Augmented Lagrangian Method for Statistical X-Ray CT Image Reconstruction," PloS one, vol. 10, p. e0140579, 2015 10.1371/journal.pone.0140579 26495975 PMC4619856

[10] Choi K., Wang J., Zhu L., Suh T. S., Boyd S., and Xing L., "Compressed sensing based cone-beam computed tomography reconstruction with a first-order method," Med Phys, vol. 37, pp. 5113–25, 9 2010 10.1118/1.3481510 20964231 PMC2945747

[11] Sidky E. Y. and Pan X., "Image reconstruction in circular cone-beam computed tomography by constrained, total-variation minimization," Phys Med Biol, vol. 53, pp. 4777–807, 9 7 2008 10.1088/0031-9155/53/17/021 18701771 PMC2630711

[12] Liu L., Lin W., and Jin M., "Reconstruction of sparse-view X-ray computed tomography using adaptive iterative algorithms," Computers in Biology and Medicine, vol. 56, pp. 97–106, 2015 10.1016/j.compbiomed.2014.11.001 25464352

[13] Liu L., Lin W., Pan J., and Jin M., "X-ray computed tomography using sparsity based regularization," Neurocomputing, vol. 173, pp. 256–69, 2016.

[14] Sidky E. Y., Kao C. M., and Pan X., "Accurate image reconstruction from few-views and limited angle data in divergent-beam CT," Journal of X-Ray Science and Technology, vol. 14, pp. 119–139, 2006.

[15] Sidky E. Y., Jørgensen J. H., and Pan X., "Convex optimization problem prototyping for image reconstruction in computed tomography with the Chambolle–Pock algorithm," Physics in Medicine and Biology, vol. 57, pp. 3065–91, 2012 10.1088/0031-9155/57/10/3065 22538474 PMC3370658

[16] Sidky E. Y., Jørgensen J. S., and Pan X., "First-order convex feasibility algorithms for x-ray CT," Medical Physics, vol. 40, p. 031115(15pp), 2013. 10.1118/1.4790698 PMC3598813 23464295

[17] Huang J., Zhang Y., Ma J., Zeng D., Bian Z., Niu S., et al, "Iterative image reconstruction for sparse-view CT using normal-dose image induced total variation prior," PloS one, vol. 8, p. e79709, 2013 10.1371/journal.pone.0079709 24260288 PMC3832537

[18] Shanzhou N., Yang G., Zhaoying B., Jing H., Wufan C., Gaohang Y., et al, "Sparse-view x-ray CT reconstruction via total generalized variation regularization," Physics in Medicine and Biology, vol. 59, p. 2997, 2014. 24842150 10.1088/0031-9155/59/12/2997 PMC4219274

[19] Liu Y., Liang Z., Ma J., Lu H., Wang K., Zhang H., et al, "Total variation-stokes strategy for sparse-view X-ray CT image reconstruction," IEEE Transactions on Medical Imaging, vol. 33, pp. 749–63, 2014 10.1109/TMI.2013.2295738 24595347 PMC3950963

[20] Yu W. and Zeng L., "ℓ_0_ Gradient Minimization Based Image Reconstruction for Limited-Angle Computed Tomography," PloS one, vol. 10, p. e0130793, 2015 10.1371/journal.pone.0130793 26158543 PMC4497654

[21] H. Zhang, H. Han, Y. Hu, Y. Liu, J. Ma, L. Li, et al., "Texture-preserving Bayesian image reconstruction for low-dose CT," 2016, pp. 97834I-97834I-6.

[22] M. Zhu and T. Chan, "An efficient primal-dual hybrid gradient algorithm for total variation image restoration," Mathematics Department, UCLA, CAM Report, pp. 08–33, 2008.

[23] Chambolle A. and Pock T., "A first-order primal-dual algorithm for convex problems with applications to imaging," Journal of Mathematical Imaging and Vision, vol. 40, pp. 120–145, 2011.

[24] Candes E. J., Romberg J. K., and Tao T., "Stable signal recovery from incomplete and inaccurate measurements," Communications on pure and applied mathematics, vol. 59, pp. 1207–1223, 2006.

[25] Donoho D. L., "Compressed sensing," IEEE Transactions on Information Theory, vol. 52, pp. 1289–1306, 2006.

[26] Stark H. and Yang Y., Vector Space Projections: A Numerical Approach to Signal and Image Processing, Neural Nets, and Optics. New Yorker: John Wiley & Sons, Inc., 1998.

[27] Jiang M. and Wang G., "Convergence of the simultaneous algebraic reconstruction technique (SART)," IEEE Trans Image Process, vol. 12, pp. 957–61, 2003 10.1109/TIP.2003.815295 18237969

[28] J. Duchi, S. Shalev-Shwartz, Y. Singer, and T. Chandra, "Efficient projections onto the l 1-ball for learning in high dimensions," in Proceedings of the 25th international conference on Machine learning, 2008, pp. 272–279.

[29] Brenner D. J. and Hall E. J., "Computed tomography—an increasing source of radiation exposure," N Engl J Med, vol. 357, pp. 2277–84, 11 29 2007 10.1056/NEJMra072149 18046031

[30] Choi K., Li R., Nam H., and Xing L., "A Fourier-based compressed sensing technique for accelerated CT image reconstruction using first-order methods," Phys. Med. Biol., vol. 59, pp. 3097–3119, 2014 10.1088/0031-9155/59/12/3097 24840019 PMC12058348

[31] Chambolle A. and Pock T., "A First-Order Primal-Dual Algorithm for Convex Problems with Applications to Imaging," J Math Imaging Vis, vol. 40, pp. 120–145, 2011.

[32] Esser E., Zhang X., and Chan T. F., "A General Framework for a Class of First Order Primal-Dual Algorithms for Convex Optimization in Imaging Science," SIAM J. IMAGING SCIENCES, vol. 3, pp. 1015–1046, 2010.

[33] Niu T. and Zhu L., "Accelerated barrier optimization compressed sensing (ABOCS) reconstruction for cone-beam CT: phantom studies," Med Phys, vol. 39, pp. 4588–98, 7 2012 10.1118/1.4729837 22830790 PMC3412436

[34] Chen G. H., Tang J., and Leng S., "Prior image constrained compressed sensing (PICCS): a method to accurately reconstruct dynamic CT images from highly undersampled projection data sets," Med Phys, vol. 35, pp. 660–3, 2 2008 10.1118/1.2836423 18383687 PMC2655145

[35] Wu D., Li L., and Zhang L., "Feature constrained compressed sensing CT image reconstruction from incomplete data via robust principal component analysis of the database," Physics in medicine and biology, vol. 58, p. 4047, 2013 10.1088/0031-9155/58/12/4047 23685849

