# Supplementary material for: Fast alternating projection methods for constrained tomographic reconstruction
Source: PLoS One. 2017 Mar 2;12(3):e0172938. doi: 10.1371/journal.pone.0172938 (PMC5416889; doi:10.1371/journal.pone.0172938)
Supplement: S1 Appendix — (DOCX) [file pone.0172938.s001.docx]

**S1 Appendix.** Convergence of *J*(**x**) using the gradient descent of TV function

The optimization of Eq. (11) is obtained using the gradient descent method with the iterative formula,

$\mathbf{x}^{k+1}=\mathbf{x}^{k}-\eta S^{k}$ ,

where *k* is the iteration number of *J*(**x**) minimization, $\eta$ is a positive step-size, *S^k^* is the normalized gradient of *J*(**x***^k^*), $S^{k}=\frac{\nabla J(\mathbf{x}^{k})}{\left\|\nabla J(\mathbf{x}^{k})\right\|}$ , and $\nabla J(\mathbf{x})=2(\mathbf{x}-\mathbf{v})+\alpha\nabla TV(\mathbf{x})$.

Here, we show that $S^{k}=\frac{\nabla J(\mathbf{x}^{k})}{\left\|\nabla J(\mathbf{x}^{k})\right\|}$ can be replaced by the gradient descent of TV function $s^{k}=\frac{\nabla TV(\mathbf{x}^{k})}{\left\|\nabla TV(\mathbf{x}^{k})\right\|}$ as Eq. (21), which can guarantee non-increasing *J*(**x**) under some condition of $\eta$, thus leading to the projection onto *C*_3_, *P*_C3_.

It can be shown that *J*(**x**) has Lipschitz continuous gradient [[5](#_ENREF_5)],

$J(\mathbf{x}^{k+1})\leq J(\mathbf{x}^{k})+\left\langle J(\mathbf{x}^{k}),\mathbf{x}^{k+1}-\mathbf{x}^{k}\right\rangle+\frac{L}{2}\left\|\mathbf{x}^{k+1}-\mathbf{x}^{k}\right\|^{2}$,

where *L* is the Lipschitz constant, whose value depends on the specified operation of 2D or 3D differentiation and can be determined numerically [[5](#_ENREF_5)].

From Eq. (21), we have $\mathbf{x}^{k+1}=\mathbf{x}^{k}-\eta s^{k}$. The inequality in Eq. (24) is converted into

$\begin{matrix} J(\mathbf{x}^{k+1})-J(\mathbf{x}^{k})\leq-\eta\left\langle2(\mathbf{x}^{k}-\mathbf{v}),\alpha\nabla TV(\mathbf{x}^{k}),s^{k}\right\rangle+\frac{L\eta}{2}^{2} \\ =-\eta\left(\left\langle2(\mathbf{x}^{k}-\mathbf{v}),s^{k}\right\rangle+\alpha\left\|\nabla TV(\mathbf{x}^{k})\right\|\right)+\frac{L\eta}{2}^{2} \end{matrix}$,

where we use the fact that $\left\|s^{k}\right\|^{2}=1$. In order to have non-increasing objective function, the right side of Eq. must be less than zero, i.e.

$-\eta\left(\left\langle2(\mathbf{x}^{k}-\mathbf{v}),s^{k}\right\rangle+\alpha\left\|\nabla TV(\mathbf{x}^{k})\right\|\right)+\frac{L\eta}{2}^{2}\leq0$.

Therefore the condition for non-increasing *J*(**x**) can be satisfied by select $\eta$ according to,

$0\leq\eta\leq T^{k}=2\left(\left\langle2(\mathbf{x}^{k}-\mathbf{v}),s^{k})\right\rangle+\alpha\left\|\nabla TV(\mathbf{x}^{k})\right\|\right)/L$.

If the sequence of *J*(**x***^k^*) approaches the local minimum, it approaches the global minimum as well since *J*(**x**) is convex. Thus, although Eq. (21) is intended for TV minimization, it can also be used to find projection **v** onto set *C*_3_, i.e. $TV(P_{C_{3}}(\mathbf{v}))\leq\tau$, given that Eq. is satisfied.

The condition of Eq. depends on the Lagrange multiplier $\alpha$, whose lower bound can be determined by Eq. (16). Indeed, the TV-POCS method used a step size that implicitly satisfied this condition (Fig 9) and led to a good reconstruction behavior.
